# Therapeutic extractions in orthodontics: An exploratory survey based study

**DOI:** 10.6026/973206300222937

**Published:** 2026-05-31

**Authors:** Sameen Faraz, Richa Sharma, Firoz A Khan, Samvit Mishra1, Purva Joneja, Megha Maheswari

**Affiliations:** 1 Department of Orthodontics and Dentofacial Orthopedics, Bhabha College of Dental Sciences, Bhopal, India; 2 Department of Dentistry, Gandhi Medical College, Bhopal, India

**Keywords:** Arch expansion, crowding, extraction decision, orthodontic treatment planning, soft tissue profile

## Abstract

Extraction versus non-extraction orthodontic treatment remains controversial due to the need to balance occlusion and facial aesthetics. Therefore, it is of interest to evaluate diagnostic aids and clinical factors in orthodonic treatment planning. Hence, a cross-sectional KAP survey was conducted among 318 orthodontists and data were analysed using Chi-square tests in SPSS. Data shows that extraction decisions were mainly influenced by crowding, soft tissue parameters, skeletal relationships and periodontal status. Thus, we report the key diagnostic and clinical determinants shaping contemporary extraction practices among Indian orthodontists.

## Background:

The decision to extract permanent teeth as part of orthodontic treatment remains one of the most debated topics in clinical orthodontics [1]. Over time, extraction and non-extraction approaches have undergone substantial shifts, largely influenced by evolving treatment philosophies, changing esthetic ideals and accumulated clinical experience. In the early years of orthodontic practice, extractions were frequently performed to manage space discrepancies and facilitate alignment of the dental arches [2]. However, Angle's philosophy of non-extraction significantly altered treatment trends by encouraging more conservative approaches, such as arch expansion and growth modification, to preserve the natural dentition whenever possible [3]. Later, Tweed, based on his clinical observations and treatment outcomes, reconsidered the role of extractions and emphasized their importance in improving facial balance, achieving optimal incisor positioning and enhancing long-term stability [4]. In the modern era of orthodontics, treatment objectives are no longer limited to achieving ideal occlusion alone; equal importance is now given to facial esthetics, soft tissue harmony and overall balance of the craniofacial complex [5]. This broader perspective has added considerable complexity to the decision-making process regarding extractions. Orthodontists are now required to carefully balance dental alignment, skeletal relationships, facial profile considerations, functional needs and long-term treatment stability before arriving at an appropriate treatment plan [6]. Consequently, the frequency of extractions has shown marked variation across different time periods, geographic regions and patient populations. Such variation reflects not only differences in esthetic preferences and technological advancements, but also the influence of individual practitioner philosophies and clinical judgment [7]. The decision to extract teeth is affected by numerous diagnostic and clinical parameters. These include the severity of crowding, sagittal and vertical skeletal relationships, incisor inclination, amount of remaining growth potential and characteristics of the soft tissue profile [8]. Additional considerations, such as periodontal status, tooth size discrepancies and the patient's age and compliance, may also influence treatment planning. Although diagnostic aids such as cephalometric analyses, study models, radiographs and clinical photographs play an essential role in supporting orthodontic diagnosis and treatment planning, the final decision in borderline cases often remains subjective and largely dependent on the clinician's interpretation, training and experience [9]. This subjectivity contributes to the diversity of treatment approaches observed among orthodontists in everyday clinical practice. Despite decades of debate and extensive clinical experience, the literature directly comparing extraction and non-extraction treatment approaches in terms of stability, facial esthetics and functional outcomes remains limited [10]. Studies specifically addressing the rationale behind clinicians' preferences and decision-making patterns are relatively scarce and universally accepted criteria for extraction do not yet exist [11]. As a result, treatment planning in orthodontics often depends on the individual clinician's judgment rather than on a standardized evidence-based framework. This lack of consensus highlights the need to better understand the factors that guide orthodontists when choosing between extraction and non-extraction approaches [12]. Therefore, it is of interest to evaluate the knowledge, attitudes and clinical parameters guiding orthodontists in the selection of extraction and non-extraction treatment approaches.

## Methodology:

This observational, descriptive, cross-sectional questionnaire-based study was conducted among registered orthodontists practicing in India. The study population comprised of orthodontists involved both in academic and clinical practice. Data was collected using a proper Knowledge, Attitude and Practice (KAP) questionnaire. The survey was distributed through online modes and offline means utilising convenience sampling approach. Online distribution was done through digital platforms, while printed copies were given wherever possible. A total of 318 orthodontists participated in the study. The sample size was determined depending on feasibility. Institutional Ethical clearance and informed consent was obtained from all participants before enrolling and blinding was maintained as responses were not revealed to anyone. The questionnaire was designed in such a manner that both qualitative and quantitative information regarding extraction decision-making is obtained. Before executing main survey, it was tested on smaller group of orthodontists to assess relevance and comprehensibility. Necessary modifications were made based on their feedback. Inclusion criteria were registered practitioners and / or academician's postgraduates in Orthodontics, Orthodontists practicing in India, irrespective of their years of clinical experience, who showed willingness to participate, were included. Exclusion criteria were orthodontists lacking valid registration, incomplete answers to questionnaires and unwilling participants. Participants from other faculties were excluded. Data was entered into excel sheets and was analyzed using SPSS software (IBM Corp) (version 26.0). Descriptive statistics were expressed as frequencies and percentages.

## Results:

Frequency and percentage distribution of the different items of questionnaire was assessed in the study. Data normality test; Kolmogorov Smirnov test was performed to determine normal distribution of the data. Chi-square test of proportion was used to assess statistically significant differences amongst the responses of questionnaire items. All statistical tests were performed at 95% confidence intervals. A p value of less than 0.05 was considered as statistically significant in the stud A total of 318 orthodontists participated in the study. The age of participants ranged from 23 to 55 years, with a mean age of 40.15 ± 9.05 years (Table 1). All participants held a postgraduate qualification (MDS) in Orthodontics. Regarding clinical experience, 42.1% of participants had 0-5 years of experience, 30.2% had 5-10 years and 27.7% had more than 10 years of experience (Table 2). Frequency and percentage distribution of the responses showing high significant on Diagnostic based Questions is shown in Table 3. Practice-related responses are summarized in Table 4. A majority of participants (73.6%) reported that periodontal implications moderately influenced their extraction decisions, while 16.7% considered it a major influence and 9.7% a minor influence (p < 0.001). In managing dental arch discrepancies caused by individual tooth shape anomalies, 81.8% preferred reshaping procedures such as interproximal reduction or restorative build-ups, whereas 15.1% opted for extraction and 3.1% considered asymmetric extraction (p < 0.001). When evaluating tooth size and arch shape, 63.8% performed quantitative analyses, 23.3% used both quantitative and qualitative methods and 12.9% relied solely on clinical examination (p = 0.0017). Most participants (80.5%) reported routinely assessing arch shape and symmetry prior to extraction decisions, while 7.5% did not and 11.9% were uncertain (p = 0.0013). Additionally, 86.8% considered vertical skeletal relationships during treatment planning, compared to 9.7% who did not and 3.5% who responded "maybe" (p = 0.003).

## Discussion:

The mean age of participants (40.15 ± 9.05 years) with 42.1% having 0-5 years of clinical experience. This age group is representative of more participation of early-career orthodontists. Literature suggests that younger orthodontists tend to show a greater preference for conservative treatment approaches, likely due to increased exposure to contemporary modalities such as temporary anchorage devices (TADs), clear aligner systems and digital treatment planning technologies during their training. This enhanced familiarity with modern orthodontic tools may partly account for the growing inclination toward non-extraction strategies, particularly in borderline cases. In the present study, 61.9% of clinicians reported that crowding of less than 4 mm influences their extraction decisions (p = 0.011). Traditionally, moderate to severe crowding (>6-8 mm) has been regarded as a primary indication for extraction therapy. However, contemporary evidence suggests that extraction decisions are no longer determined solely by arch length discrepancy. Increasingly, clinicians incorporate soft tissue balance and incisor positioning into their treatment planning considerations. A recent systematic review and meta-analysis by Elias *et al.*(2024) [3] found that premolar extraction therapy was associated with a reduction in intermolar width and significant lip retraction compared to non-extraction treatment, although overall occlusal outcomes and PAR scores were similar between groups. These findings highlight that the degree of crowding alone should not dictate extraction decisions. The lower crowding threshold observed in our survey may indicate a more preventive and profile-conscious approach, where clinicians aim to control incisor protrusion and minimize soft tissue strain rather than waiting for more severe discrepancies to develop. Soft tissue parameters emerged as key determinants in treatment planning. A majority of respondents considered a nasolabial angle of 95-100° clinically significant (64.8%, p = 0.002) and relied on Steiner's S-line for lip assessment (69.2%, p = 0.021). Lip thickness was also identified as an important factor by 67.9% of participants (p = 0.001). This strong emphasis on soft tissue harmony reflects contemporary orthodontic philosophy, which prioritizes facial esthetics alongside occlusal correction. Although premolar extractions have been shown to produce measurable lip retraction, particularly in cases of maxillary protrusion, the extent of profile change is often modest and largely influenced by the initial lip strain and underlying skeletal pattern. The predominant reliance on the S-line in our study supports its continued clinical relevance, while other reference lines such as the E-line may still be valuable in population-specific assessments [13]. Vertical and occlusal considerations also played a significant role. Most participants regarded a 2-4 mm curve of Spee as relevant in extraction planning (62.9%, p = 0.003) and 86.8% routinely evaluated vertical skeletal relationships (p = 0.003). A pronounced curve of Spee and vertical discrepancies can increase anchorage requirements and heighten the risk of uncontrolled incisor proclination during non-extraction levelling [14]. In selected hyperdivergent cases, extraction therapy may allow for more controlled biomechanics. Nevertheless, current literature does not conclusively demonstrate superior vertical control with extraction therapy alone [15]. Strategic extractions may reduce strain on compromised tissues and potentially enhance long-term stability, whereas excessive expansion in a vulnerable periodontium may increase the risk of dehiscence or gingival recession [16]. A notable observation was the strong preference (81.8%) for interproximal reduction (IPR) in cases of tooth size discrepancy. This finding reflects the increasing adoption of minimally invasive orthodontic strategies, where selective enamel reduction can create limited space without resorting to extractions. When carefully performed with proper finishing and fluoride application, IPR does not appear to significantly elevate caries risk [17]. Similarly, 72.3% of respondents favoured arch expansion when patients were reluctant to undergo extractions. Skeletal anchorage-supported expansion has been reported to reduce the need for extractions in selected cases, although long-term stability remains dependent on skeletal pattern, growth potential and appropriate case selection. Only 24.8% of participants considered impacted third molars a contraindication to non-extraction therapy. This observation is consistent with longitudinal studies demonstrating minimal association between third molars and late anterior crowding. While extraction therapy may improve third molar angulation, its overall clinical significance appears limited. Despite identifiable trends, variability in threshold values underscores the absence of universally accepted criteria for extraction decisions. Current comparative evidence between extraction and non-extraction approaches remains largely retrospective and heterogeneous. Emerging artificial intelligence-based predictive models show promising accuracy in estimating extraction needs; however, these technologies should be regarded as adjunctive tools that support rather than replace clinical judgment [18].

## Conclusion: 

Therapeutic extraction decisions among orthodontists are based on a multifactorial diagnostic approach that integrates skeletal, dental, soft-tissue and periodontal considerations rather than a single factor. Severity of crowding remains a major determinant, while soft-tissue parameters, vertical skeletal relationships and arch form; symmetry and midline discrepancies also play significant roles in treatment planning. Although extraction remains a relevant evidence-based option, contemporary orthodontic practice increasingly favors individualized and minimally invasive alternatives such as interproximal reduction and arch expansion whenever appropriate.

## Figures and Tables

**Table 1:** Descriptive statistics of the age of the study participants

	**N**	**Minimum**	**Maximum**	**Mean**	**Std. Deviation**
Age (in years)	318	23	55	40.15	9.05

**Table 2:** Frequency and percentage distribution of the study participants according to years of clinical experience

**Years of Clinical Experience**		**Frequency(n)**	**Percentage(%)**
	0-5 years	134	42.1
	5-10 years	96	30.2
	10+ years	88	27.7
	Total	318	100

**Table 3:** Frequency and percentage distribution of the responses showing high significant on diagnostic based questions

**Questions and their responses**		**Frequency(n)**	**Percentage(%)**	**p value**
What is the minimum degree of nasolabial angle that influences your decision for extractions?	90-95 degree	64	20.1	0.002*
	95-100 degree	48	15.1	
	100-105 degree	206	64.8	
What is the minimum degree of crowding (in mm) that typically influences your decision for extractions?	< 4mm	19	5.9	0.011*
	4-8 mm	79	24.8	
	> 8mm	220	69.1	
What specific role does upper lip thickness play in your decision to extract teeth?	12-14 mm	51	16	0.001*
	14-15 mm	51	16	
	>15 mm	216	67.9	
Which of the following parameter do you rely on as extraction decision accordance to lip prominence	H line angle	38	11.9	0.021*
	Lip relation to S line	220	69.2	
	Lip relation to Rickets E line	60	18.9	
Which threshold measurement for the curve of Spee influences your decision for extraction?	2-4 mm	50	15.7	0.003*
	4-5 mm	68	21.4	
	>6 mm	200	62.9	
Do you evaluate arch shape and symmetry before deciding on extractions?	Yes	256	80.5	0.0013*
	No	24	7.5	
	Maybe	38	11.9	
Do you consider vertical skeletal relationships when planning extractions?	Yes	276	86.8	0.003*
	No	31	9.7	
	Maybe	11	3.5	
Do you routinely assess dental midline discrepancies during treatment planning?	Yes	263	82.7	0.001*
	No	54	17	
	Maybe	1	0.3	
How do you evaluate the interplay between tooth size and arch length when deciding on extractions?	Perform quantitative analyses (e.g., Bolton, arch length discrepancy)	203	63.8	0.0017*
	Assess qualitatively during clinical examination	41	12.9	
	Use both methods equally	74	23.3	

**Table 4:** Frequency and percentage distribution of the responses showing high significance on clinical approach-based question

**Questions and their responses**		**Frequency (n)**	**Percentage(%)**	**p value**
How do you handle dental arch discrepancies caused by individual tooth shape anomalies?	Always extract	48	15.1	0.0001*
	Attempt reshaping (e.g., IPR, build-ups)	260	81.8	
	Asymmetric extraction	10	3.1	
What would be your level of influence of periodontal implications on extraction decision?	Major	234	73.6	0.0001*
	Moderate	53	16.7	
	Minor	31	9.7	
In case of all four premolar extractions in conjunction with deeply carious first molar, what would be your preferred treatment plan?	Extraction of first molar	260	81.8	0.0017*
	Endodontically preserving first molar	58	18.2	
In a case of definite extraction with a patient non-compliant for extraction, what would be your preferred treatment plan?	Increased IPR	49	15.4	0.0002*
	Arch expansion	230	72.3	
	Change of mechanotherapy	39	12.3	
Do you believe that the presence of impacted third molars is a barrier for non-extraction treatment?	Yes	79	24.8	0.004*
	No	218	68.6	
	Maybe	21	6.6	
